# Serotyping of sub-Saharan Africa *Salmonella* strains isolated from poultry feces using multiplex PCR and whole genome sequencing

**DOI:** 10.1186/s12866-021-02085-6

**Published:** 2021-01-19

**Authors:** Assèta Kagambèga, Lari M. Hiott, David S. Boyle, Elizabeth A. McMillan, Poonam Sharma, Sushim K. Gupta, Hazem Ramadan, Sohyun Cho, Shaheen B. Humayoun, Tiffanie A. Woodley, Nicolas Barro, Charlene R. Jackson, Jonathan G. Frye

**Affiliations:** 1 Bacterial Epidemiology and Antimicrobial Resistance Research Unit, U.S. National Poultry Research Center, USDA, ARS, Athens, GA USA; 2 Laboratoire de Biologie Moléculaire, d’épidémiologie et de surveillance des bactéries et virus transmissibles par les aliments (LaBESTA)/Ecole Doctorale Sciences et Technologies (EDST)/Université Joseph KI-ZERBO, Ouagadougou, Burkina Faso; 3 Institut des Sciences, Ministère des enseignement supérieur, de la recherche scientifique et de l’innovation, Ouagadougou, Burkina Faso; 4 PATH, Suite 200, Seattle, WA 98121 USA; 5 Hygiene and Zoonoses Department, Faculty of Veterinary medicine, Mansoura University, Mansoura, 35516 Egypt

**Keywords:** *Salmonella*, Serotyping, Molecular method

## Abstract

**Background:**

*Salmonella enterica* remains a leading cause of food-borne diseases worldwide. Serotype information is important in food safety and public health activities to reduce the burden of salmonellosis. In the current study, two methods were used to determine serotypes of 111 strains of *Salmonella* isolated from poultry feces in Burkina Faso. First, *Salmonella* Multiplex Assay for Rapid Typing (SMART) Polymerase Chain Reaction (PCR) was used to determine the serovars of the *S. enterica* isolates. Second, serovar prediction based on whole genome sequencing (WGS) data was performed using SeqSero 2.0.

**Results:**

Among the 111 *Salmonella* isolates, serotypes for 17 (15.31%) isolates were identified based on comparison to a panel of representative SMART codes previously determined for the 50 most common serovars in the United States. Forty-four (44) new SMART codes were developed for common and uncommon serotypes. A total of 105 (94.59%) isolates were serotyped using SeqSero 2.0 for serovar prediction based on WGS data.

**Conclusion:**

We determined that SeqSero 2.0 was more comprehensive for identifying *Salmonella* serotypes from Burkina Faso than SMART PCR.

## Background

The discovery of *Salmonella* was made by Theobald Smith in 1855 from the intestines of a pig suffering from swine fever [1, 2]. *Salmonella* is a genus of gram-negative bacteria in the family of Enterobacteriaceae with two species: *Salmonella bongori* and *Salmonella enterica.* The species *S. enterica* includes more than 2579 serovars and is a major cause of food-borne illness in humans [3, 4]. The subspecies of *S. enterica* are *enterica, salamae, arizonae, diarizonae, houtenae*, and *indica* [5]. *Salmonella enterica subsp. enterica* includes over 1400 serotypes and causes approximately 99% of *Salmonella* infections in humans and warm-blooded animals [6]. *Salmonella* serovars Typhi, Paratyphi A, and B cause enteric fever, a systemic febrile illness that only occurs in humans. Non-typhoidal *Salmonella* (NTS) infect a variety of hosts including warm blood animals. NTS are one of the leading causes of bacterial diarrhea worldwide, but the majority of cases occur in Sub-Saharan Africa [7]. Human disease can result from exposure to many sources such as infected animals, contaminated foodstuffs, contaminated water, and direct contact with infected environment or directly between humans.

In Sub-Saharan Africa, the socio-economic burden of NTS is difficult to quantify due to the lack of a standard method of assessment, which is compounded by under-reporting in many cases [8–10]. NTS serotype identification is important for the control of foodborne disease incidence. The traditional method for *Salmonella* serotyping is the slide and tube agglutination tests using the O and H antigen according to Kauffman-White scheme [11, 12]. However, this method is not always accessible in low- and middle-income countries (LMICs) because the anti-sera used for agglutination is very expensive and some stocks are often not available. In many LMICs like Burkina Faso, only antisera for the agglutination of Vi antigen is available for the detection of *Salmonella* Typhi and Paratyphi A or B in hospitals. A few studies have investigated *Salmonella* serotyping using the Kauffman-White scheme in Burkina Faso by collaborating with laboratories in industrialized countries [13–16]. Studies in other Sub-Saharan African countries, such as Rwanda and The Central African Republic, and have also utilized traditional serotyping through collaboration to investigate NTS from human clinical sources [17, 18].

To establish the true incidence of NTS diseases in developing countries, development of new, cheaper methods is needed for *Salmonella* serotyping.

Molecular methods are well suited for this because their specificity is comparable with traditional Kauffman-White serotyping. However, many of these modern detection methods for *Salmonella* are still absent in some developing countries, particularly in Burkina Faso. Countries with limited financial resources cannot implement well-established yet complex nucleic acid analysis systems or laboratory-developed tests through a network of centralized laboratories. These molecular methods require specific and complex equipment, sensitive reagents, dedicated infrastructure, and deep technical knowledge, which are not available in many LMICs. Therefore, it is often a necessity for researchers from LMICs to collaborate with high income countries and test available modern techniques for *Salmonella* serotyping to determine if the method can be adapted for their country.

In this study, the high-throughput molecular determination of *Salmonella enterica* serovars by use of *Salmonella* Multiplex Assay for Rapid Typing (SMART) PCR using capillary electrophoresis and whole genome sequencing (WGS) were compared to determine their accuracy in identifying serotypes of NTS isolated from Burkina Faso. The SMART method was developed by Leader et al. [12] for discrimination of most common serotypes that are reported in the United States based upon their genetic differences. The genotypic serotype prediction from WGS data was done using SeqSero 1 and 2 [19, 20].

## Methods

### Bacterial strains

The 111 isolates used in this study were obtained from the Laboratoire de Biologie Moléculaire, d’épidémiologie et de surveillance des bactéries et virus transmissible par les aliments (LaBESTA)/Université Joseph KI-ZERBO, Burkina Faso. The strains were isolated from poultry feces and the serotype of each confirmed following the methodologies described in the International Organization for Standardization 6579–2017 [21].

### High-throughput molecular determination of *Salmonella enterica* serovars

We used the SMART method developed by Leader et al. [12] with slight modification. *Salmonella* strains were streaked onto blood agar and incubated for 18–20 h at 36 °C. Then, one colony from each plate was cultured in 5 mL of Luria Bertani (LB) broth, (Difco™, Becton Dickinson and Company, Sparks, MD) and incubated for 18 h at 37 °C with shaking. The genomic DNA was then isolated from the overnight culture using the GenElute bacterial genomic DNA kit (Sigma-Aldrich, St. Louis, MO, USA) and following the kit instructions for use. Once extractions were completed, the DNA was analyzed on the Nanodrop 2000 for DNA quality measuring the 260/280 nm. All DNA were then stored at − 20 °C until ready for PCR and library preparation.

### PCR amplification

Each PCR mixture contained 12.5 μL of Immolase DNA polymerase 2X master mix (Bioline, Inc., Randolph, MA, USA), 2.5 μL of 10X primer master mix, 3 mM MgCl_2_, and 1 μL of extracted DNA with addition of nuclease free water to a final reaction volume of 25 μL. The cycling conditions used in the thermal cycler were 94 °C for 10 min; 25 cycles of 94 °C for 30 s, 57 °C for 90 s, and 72 °C for 30 s; 72 °C for 5 min; 15 cycles of 94 °C for 30 s, 68 °C for 90 s, and 72 °C for 30 s; and 72 °C for 5 min. For each run, the negative control was sterile water and positive controls were genomic DNA from *Salmonella* Typhimurium LT2, *S.* Typhi CT18, *S.* Enteritidis strains 21,027 and 98,104. The primers used in this study were previously described by Leader et al. [12]. The amplicon samples were then diluted and analyzed on an ABI 3130XL Genetic Bioanalyzer using capillary electrophoresis.

Genemapper software v3.5 (Applied Biosystems, Foster City, CA, USA) was used to analyze the sizes of resulting PCR products according to the protocol developed by Leader et al. [12]. Scoring was based upon the presence of a PCR product that corresponded to the predicted amplicon size, as detected in control reactions with DNA from *S.* Typhimurium, *S.* Typhi, and *S.* Enteritidis. Each PCR product detected was given a number (1 through 16) according to the size of the amplicon [12]. The amplicons detected for each isolate were combined to create a SMART code that corresponds to serotypes previously screened by this method.

### Whole genome sequencing of *Salmonella* strains

Extracted DNA was quantified using the Qubit double-strandedDNA high-sensitivity assay kit according to the manufacturer’s instructions (Life Technologies Corp., Carlsbad, CA, USA). The Illumina libraries were prepared using the Nextera XT DNA library preparation kit and Nextera XT index primers (Illumina, san Diego, CA, USA). The library fragment size distribution was checked using the Bioanalyzer 2100 with an Agilent HS DNA kit (Agilent Technologies, Santa Clara, CA,USA) and quantified using a Qubit DNA HS assay kit in a Qubit fluorometer (Thermo Fisher Scientific, Waltham, MA, USA). The generated libraries were then sequenced using a MiSeq version 2 reagent kit (Illumina) with 500 and 300 cycles. The paired-end read length of 2 X 250 bp was used for 500 cycles and 2 X 150 bp for 300 cycles on the MiSeq platform (Illumina). The quality metrics of the reads were performed by FastQC (http://www.bioinformatics.babraham.ac.uk/projects/fastqc/). The sequences were then assembled using the A5-miseq assembler [22], and deposited into NCBI under BioProject no. PRJNA679582 (https://www.ncbi.nlm.nih.gov/bioproject/PRJNA679582). The genome sequence was annotated via the NCBI Prokaryotic Genome Annotation Pipeline [23].

### Serovar prediction from WGS using in silico SeqSero tool

SeqSero version 2.0 was used to determine the serotype of the 111 *Salmonella* isolates [17].

## Results

### The SMART PCR generated some new codes different to those found in the United States

One hundred and eleven (111) samples were analyzed to determine the serotypes of *Salmonella* strains isolated from Burkina Faso using the SMART PCR database developed for the 50 most common *Salmonella* serovars found in the U.S. [12]. Among the 111 *Salmonella* isolates, 17 (15.31%) serotypes were identified based on comparison to the panel of SMART codes. Forty-four isolates were assigned new codes not included in the SMART PCR database (Table 1). The serovars Enteritidis, Agona, Virchow, Poona, and Liverpool did not generate any new codes. However, serovars Typhimurium, Duesseldorf, Tennessee, Gaminara, and Schwarzengrund developed at least one new code in addition to the original (Table 1). New SMART codes were determined for some uncommon serotype including Bredeney, Hato, Brancaster, Kaapstad, Amoutive, and others (Table 2).

**Table 1:** SMART-PCR and SeqSero results

Sample ID	Serogroup	SMART PCR code	Serotype by SMART	Antigenic serotype by SeqSero2	Serotype by SeqSero2
S38	C2-C3	1–2–5-6-11	Dusseldorf	8:z4,z24:-	Albany or Duesseldorf
S39	B	1–5–7-9-11-16	new code	4:e,h:e,n,x	Chester
S40	E1	1–2–5-7-11-13	new code	3,10:z29:-	Jedburgh
S47	B	1–2–5-6-10-11	new code	4:g,m,s:-	Hato
S52	B	1–5–7-9-11-16	new code	4:i:1,2	Typhimurium
S53	B	1–2–5-6-11	new code	4:g,m,s:-	Hato
S58	G	1–5–6-7-11-16	Telelkebir	13:d:e,n,z15	Telelkebir
S59	B	1–2–5-6-7-8-11-12-13-14-16	Typhimurium	4:i:1,2	Typhimurium
S60	G	1–5–6-7-9-11-16	new code	13:d:e,n,z15	Telelkebir
S61	D1	1–2–3-5-6-11-14-15	Enteritidis	9:g,m:-	Enteritidis
S62	D1	1–2–3-5-6-11-14-15	Enteritidis	9:g,m:-	Enteritidis
S63	B	1–2–5-6-11	new code	4:g,m,s:-	Hato
S64	B	1–2–5-11-13	Agona	4:f,g,s:-	Agona
S65	B	1–2–5-6-11-13	new code	4:f,g:-	Derby
S66	E4	1–2–5-6-11-13-16	new code	1,3,19:f,g:1,5	N/A
S67	B	1–5–7-9-11-16	new code	4:e,h:e,n,x	Chester
S68	B	1–2–5-6-10-11	new code	4:g,m,s:-	Hato
S69	C2-C3	1–2–5-10-11-13-16	new code	4:i:1,2	Typhimurium
S70	B	1–2–5-6-11	new code	4:f,g:-	Derby
S71	C1	1–5–7-11-13-16	Virchow	7:r:1,2	Virchow
S72	M	1–2–5-11-13-16	new code	28:d:1,5	Amoutive
S74	C2-C3	1–2–5-10-11-13-16	new code	8:i:z6	Kentucky
S75	B	1–2–5-6-7-8-11-12-13-14-16	Typhimurium	4:i:1,2	Typhimurium
S80	B	1–2–5-6-10-11	new code	4:f,g:-	Derby
S82	B	1–5–7-9-11-16	new code	4:e,h:e,n,x	Chester
S83	B	1–2–5-6-7-8-11-12-13-14-16	Typhimurium	4:i:1,2	Typhimurium
S86	B	1–2–5-6-7-10-11-13	new code	4:z29:-	Brancaster
S90	I	1–5–6-10-11-16	Gaminara	16:d:1,7	Gaminara
S91	B	1–2–5-6-7-10-11-13	new code	16:d:1,8	Gaminara
S92	B	1–2–5-6-7-10-11-13	new code	4:f,g:-	Derby
S93	B	1–5–6-7-11-16	Schwarzengrund	4:d:1,7	Schwarzengrund
S94	B	1–2–5-6-11-13	new code	4:f,g:-	Derby
S96	Poly D	1–5–6-7-11-13-16	new code	4:z29:-	Brancaster
S97	B	1–2–5-6-11-13	new code	4:f,g:-	Derby
S99	E1	1–2–5-6-7-11-16	new code	3,10:e,h:1,6	Anatum
S102	B	1–5–6-7-10-11-16	Bredeney	4:l,v:1,7	Bredeney
S103	E4	1–5–7-11-16	Liverpool	4:f,g:-	Derby
S104	polyE	1–2–5-6-9-11	new code	47:z38:-	Alexanderplatz
S105	B	1–2–5-6-7-10-11-13	new code	4:f,g:-	Derby
S106	C2-C3	1–2–5-10-11-16	new code	8:i:z6	Kentucky
S107	B	1–5–6-7-10-11-16	Bredeney	4:l,v:1,7	Bredeney
S108	C1	1–2–5-11-13	new code	7:z29:-	Tennessee
S109	B	1–2–5-6-7-10-11-13	new code	4:f,g:-	Derby
S110	D1	1–3–5-7-10-11-16	new code	9:e,h:1,5	Eastbourne
S111	C1	1–2–5-11-13	new code	7:z29:-	Tennessee
S112	B	1–2–5-6-8-11-13	new code	4:g,m,s:-	Hato
S114	B	1–2–5-6-8-11-13	new code	4:g,m,s:-	Hato
S115	B	1–2–5-6-11-13	new code	4:f,g:-	Derby
S118	G	1–6–7-9-11-16	Poona	13:z:1,6	Poona
S120	B	1–2–5-6-7-10-11-13	new code	4:f,g:-	Derby
S121	G	1–6–7-9-11-16	Poona	13:z:1,6	Poona
S123	B	1–5–6-7-10-11-16	Bredeney	4:l,v:1,7	Bredeney
S124	B	1–2–5-6-11-13	new code	4:f,g:-	Derby
S125	B	1–2–5-6-8-11-13	new code	4:g,m,s:-	Hato
S126	C2-C3	1–2–5-10-11-16	new code	8:i:z6	Kentucky
S132	B	1–2–5-6-7-10-11-13	new code	4:f,g:-	Derby
S133	B	1–2–5-6-7-10-11-13	new code	4:f,g:-	Derby
S140	PolyE	1–2–5-6-9-11	new code	47:z38:-	Alexanderplatz
S143	PolyE	1–2–5-6-9-11	new code	47:z38:-	Alexanderplatz
S145	C1	1–2–5-11-13	new code	7:z29:-	Tennessee
S146	C1	1–2–5-6-10-11-13	Tennessee	7:z29:-	Tennessee
S147	PolyE	1–5–6-7-9-11-16	new code	47:l,v:e,n,x	Drac
S148	E1	1–5–6-7-10-11-16	new code	3,10:e,h:1,5	Muenster
S149	E1	1–5–6-7-10-11-16	new code	3,10:e,h:1,6	Muenster
S150	E1	1–5–6-7-9-11-16	Muenster	3,10:e,h:1,5	Muenster
S151	E1	1–5–6-7-9-11-16	Muenster	3,10:e,h:1,6	Muenster
S152	D1	1–5–6-7-9-11-16	Muenster	3,10:e,h:1,5	Muenster
S153	E1	1–5–6-7-9-11-16	Muenster	3,10:e,h:1,5	Muenster
S154	E1	1–5–6-7-10-11-16	new code	3,10:e,h:1,5	Muenster
S155	G	1–7–9-10-11-16	new code	39:f,g:e,n,z15	N/A
S156	G	1–7–9-10-11-16	new code	13:z:1,6	Poona
S162	E4	1–5–6-7-11-13	new code	1,3,19:b:-	N/A
S163	B	1–2–5-6-11-13	new code	4:f,g:-	Derby
S164	E4	1–5–6-7-11-13	new code	1,3,19:b:-	N/A
S165	B	1–2–5-6-11-13	new code	4:f,g:-	Derby
S167	E4	1–5–6-7-11-13	new code	1,3,19:b:-	N/A
S168	B	1–2–5-6-7-8-11-12-13-14-16	Typhimurium	4:i:1,2	Typhimurium
S169	B	1–2–5-6-7-10-11	new code	4:g,m,s:-	Hato
S170	B	1–2–5-6-11-13	new code	4:f,g:-	Derby
S171	B	1–2–5-6-10-11-13	new code	7:z29:-	Tennessee
S172	C2-C3	1–2–5-10-11-13-16	new code	8:i:z6	Kentucky
S183	B	1–2–5-6-11-13	new code	4:f,g:-	Derby
S184	B	1–2–5-6-11	new code	4:g,m,s:-	Hato
S185	B	1–2–5-6-11	new code	4:g,m,s:-	Hato
S186	B	1–2–5-6-11	new code	4:g,m,s:-	Hato
S187	B	1–2–4-5-6-11	new code	4:g,m,s:-	Hato
S188	B	1–2–5-6-11	new code	4:g,m,s:-	Hato
S191	B	1–2–5-6-11	new code	4:g,m,s:-	Hato
S194	B	1–2–5-6-11	new code	4:g,m,s:-	Hato
S196	B	1–2–5-6-11	new code	4:g,m,s:-	Hato
S197	B	1–2–5-6-11	new code	4:g,m,s:-	Hato
S198	B	1–2–5-6-11-13	new code	4:f,g:-	Derby
S199	B	1–2–5-6-11-13	new code	4:f,g:-	Derby
S200	B	1–2–5-6-11	new code	4:g,m,s:-	Hato
S201	B	1–2–5-6-11	new code	4:g,m,s:-	Hato
S202	B	1–2–5-6-11	new code	4:g,m,s:-	Hato
S203	B	1–2–5-6-11	new code	4:g,m,s:-	Hato
S204	B	1–2–5-6-11	new code	4:g,m,s:-	Hato
S207	B	1–2–5-6-11	new code	4:g,m,s:-	Hato
S208	B	1–2–5-6-11	new code	4:g,m,s:-	Hato
S209	B	1–2–5-6-11	new code	4:g,m,s:-	Hato
S212	B	1–2–5-6-11	new code	4:g,m,s:-	Hato
S216	B	1–2–5-6-11	new code	4:g,m,s:-	Hato
S219	B	1–2–5-6-11	new code	4:g,m,s:-	Hato
S248	B	1–2–5-6-10-11	new code	4:f,g:-	Derby
S249	B	1–2–5-6-10-11	new code	4:f,g:-	Derby
S251	B	1–2–5-6-10-11-13	new code	4:f,g:-	Derby
S252	C1	1–2–5-11-13	new code	7:z29:-	Tennessee
S253	E4	1–5–6-7-11-13	new code	1,3,19:b:-	N/A
S254	B	1–2–5-10-11-13-16	new code	8:i:z6	Kentucky
S255	C2-C3	1–2–5-10-11-13-16	new code	4:i:1,2	Typhimurium

**Table 2:** New SMART codes generated by Sub-Saharan African salmonella serotypes as compared to existing serotype specific codes

Serotypes	New SMART codes	Exisiting SMART code from Leader et al. 2009
Hato	1–2–5-6-11	None
	1–2–5-6-8-11-13	
	1–2–5-6-10-11	
	1–2–5-6-7-10-11	
	1–2–4-5-6-11	
Typhimurium	1–5–7-9-11-16	1–2–5-6-7-8-11-12-13-14-16
	1–5–6-7-11-16	
	1–2–5-10-11-13-16	
Kentucky	1–2–4-5-6-10-11-13-16	1–5–6-9-11
	1–2–5-11-16	
	1–2–5-10-11-13-16	
	1–2–5-10-11-16	
Derby	1–2–5-6-11-13	1–2–5-6-7-11-14
	1–2–5-6-11	
	1–2–5-6-7-8-11-12-13-14-16	
	1–2–5-6-10-11	
	1–2–5-6-7-10-11-13	
	1–2–5-6-11-13	
	1–2–5-6-11	
	1–2–5-6-10-11	
	1–2–5-6-10-11-13	
Muenster	1–5–6-7-10-11-16	1–5–6-7-9-11
Schwarzengrund	1–5–6-11-16	1–5–6-7-11
Tennessee	1–2–5-11-13	None
Telelkebir	1–5–6-7-9-11-16	1–5–6-7-11
Brancaster	1–2–5-6-11-13	None
	1–2–5-6-7-10-11	
	1–2–5-6-7-10-11-13	
	1–5–6-7-11-13-16	
1,3,19:b:-	1–5–6-7-11-13	None
1,3,19:f,g:1,5	1–2–5-6-11-13-16	None
39:z10:e,n,z15	1–2–4-5-10-11-13-16	None
	1–7–9-10-11-16	
Albany or Duesseldorf	1–2–5-6-11	None
Chester	1–5–7-9-11-16	1–5–7-11
Jedburgh	1–2–5-7-11-13	None
Amoutive	1–2–5-11-13-16	None
Gaminara	1–2–5-6-7-10-11-13	None
	1–2–5-6-11-13	
Anatum	1–2–5-6-7-11-16	1–2–5-6-7-11-12
Alexanderplatz	1–2–5-6-9-11	None
Eastbourne	1–3–5-7-10-11-16	None
Poona	1–7–9-10-11-16	1–6–7-9-10-11
Drac	1–5–6-7-9-11-16	None

### SeqSero method for determination of Salmonella serovars using whole genome sequencing data

We performed WGS on the 111 *Salmonella* isolates. Using SeqSero 2 we determined the serotype of 105 (94.59%) *Salmonella* isolates. Twenty-six different serotypes were identified: Enteritidis, Typhimurium, Kentucky, Agona, Virchow, Anatum, Derby, Hato, Chester, Jedburgh, Schwarzengrund, Tennessee, Albany, Duesseldorf, Poona, Eastbourne, Gaminara, Drac, Alexanderplatz, Brancaster, Bredeney, Amoutive, Telelkebir, Liverpool, Muenster, Monschaui. Among the serotypes predicted, *S*. *Hato* was the most frequently identified serotype with 28 of the 111 isolates (25.22%), followed by Derby (20.72%), Typhimurium (6.30%), Muenster (6.30%), Tennessee (5.40%), and Kentucky (4.50%) (Table 1).

SMART PCR assigned some isolates with new codes without serotype predictions and SeqSero assigned them a serotype. For example, SMART PCR predicted serotype Dusseldorf for one isolate and SeqSero predicted Albany or Dusseldorf because both share the same antigenic profile “8:z4,z24:-”(Table 1).

## Discussion

Serotyping is an important tool for monitoring for foodborne outbreaks and in understanding the diversity and distribution of serotypes within populations, flocks, and herds. However, serotyping by traditional methods remains inaccessible for many LMICs. In this study we investigated two molecular serotyping method to identifying serotypes for 111 isolates from Burkina Faso. The goal was to serotype *Salmonella enterica* using rapid and accessible molecular methods as opposed to immunologic approaches to antigen characterization. The traditional method of serotyping using the Kauffman-White Scheme is expensive, time consuming, and training is needed to accurately read results. The SMART PCR is faster and cheaper than traditional serotyping and can be automated to read results. For example, it is possible to test two 96-well plates of *Salmonella* strains in one SMART PCR run. The reagents required are also less expensive than antisera and available from many different vendors worldwide [12]. Both factors would benefit outbreak control in the developing word. In the present study, 44 isolates were assigned new codes not previously included in the SMART PCR database. This result will be benefit LMICs by extending the original SMART code database with serotypes more prevalent in other part of the world, particularly from Sub-Saharan Africa. This could be used in future studies to analyze the diversity of *Salmonella* serotypes. Moreover, *Salmonella* infections can globally circulate and a serotype from another region can potentially emerge as a common serotype persistent in other places than from were first reported. For example, Wong et al. [24] demonstrated that a Multidrug Resistant (MDR) S. Typhi H58 emerged in South Asia was propagated to many locations around the world, including countries in Southeast Asia, Western Asia and East Africa.

However, a limitation of SMART PCR is the identification of new codes without an assigned serotype. Previously pulsed-field gel electrophoresis patterns were used to compliment SMART codes. The SMART assay was initially developed to identify the 50 most common serotypes from clinical isolates found in the Northwestern USA [12]. Our study identified many new SMART codes associated with uncommon serotypes and there is an urgent need to extend the database to include more *Salmonella* serotypes as classified by the Kauffmann -White scheme [25]. This will greatly increase the usability of the SMART PCR around the world. The original SMART codes should be renewed every five years because *Salmonella* infections are in constant flux and any serotype can emerge as a top serotype at any time. Moreover, the capillary electrophoresis machine is very sensitive to power surges and needs to be protected appropriately, which is a challenge for laboratories in developing countries. Furthermore, the widespread application of NGS tools will at some point render capillary electrophoresis redundant.

### SeqSero 2

SeqSero 2.0 is a new software tool for *Salmonella* serotype determination from WGS data described by Zang et al. [20]. SeqSero 2.0 identifies *Salmonella* serotypes with more precision in comparison to the first edition of SeqSero [19]. We initially used SeqSero 1.0 and found many strains with unknown serotypes. When we used SeqSero 2.0 there was a significant improvement in serotyping results (data not shown). Therefore, we can say that SeqSero 2.0 is a very powerful tool for determining serotypes using WGS data. However, this tool must be constantly updated to consider new serotypes that are identified by the Kauffman-White scheme [25]. In the present study, SeqSero 2.0 was able to predict 94.59% of the serotypes from the submitted strains. This result agrees with the results found by Banerji et al. [26]; SeqSero 2.0 was able to accurately predict most serotypes but not all. As serotyping based on WGS becomes the new gold stand, it will be important to resolve serotypes to a single correct identification.

SeqSero 2.0 has many advantages for *Salmonella* serotyping and fewer limitations as compared to SMART PCR. SeqSero 2.0 uses assembled whole genomes and returns a result of an antigenic profile with the serotype name in a few minutes. In the present study, we were able to predict 105 Salmonella serotypes from 111 submitted (94.59%) using SeqSero 2.0. However, using SMART PCR, only 17 of 111 strains analyzed (15.31%) were assigned a serotype. These findings demonstrate the limitation of SMART PCR assays for *Salmonella* strains isolated outside the U.S. The original SMART codes were created using only clinical isolates from the North Western USA, in this current study there are differences not only in the geographic region but also in using predominantly veterinary and food related isolates which may have a bearing on the significant variation in SMART codes.

Whole genome sequencing is a powerful tool for understanding *Salmonella* epidemiology and distribution of disease. However, sequencing is very expensive, time consuming, and requires data storage capacities and staff with high technical and bioinformatic skills. SeqSero 2 analysis of some isolates provided two possible serotypes sharing the same antigenic profile (or formula) but with differing minor O antigenic factors or in other cases gave no serotype. These unpredicted serovars are either not included in the SeqSero database and could be shared as part of the iterative construction of the serotype database for use in the next version 3.0.

In this study we noticed that SeqSero and SMART PCR can be complementary for the determination of certain serotypes. For example, SMART PCR predicted some isolates as *S.* Duesseldorf, and these same isolates were predicted by SeqSero 2 as *S.* Albany or Duesseldorf.

## Conclusions

*Salmonella* epidemiology is a worldwide public health problem. In this study, the results highlight the accuracy of modern molecular methods and in doing so also the need of less expensive methods for rapid serotyping of *Salmonella* in developing countries. SeqSero 2.0 is very accurate for *Salmonella* serotyping, but WGS is very expensive, especially for LMICs and a role for NGS may be as a shared resource with other needs such as antimicrobial drug resistance, a growing risk to public health with grave consequences. Therefore, researchers should continue developing less expensive and accurate methods of *Salmonella* serotyping that can be accessible worldwide. However, both methods require a clonal culture isolate and so while these molecular tools offer great accuracy, the need for classical microbiology cannot be overlooked in first culturing and identifying the pathogen from its sample matrix.

## Data Availability

The datasets and Accession numbers generated during the current study are available in the [NCIBI] repository under BioProject no. PRJNA679582 [https://www.ncbi.nlm.nih.gov/bioproject/PRJNA679582;https://www.ncbi.nlm.nih.gov/biosample?Db=biosample&DbFrom=bioproject&Cmd=Link&LinkName=bioproject_biosample&LinkReadableName=BioSample&ordinalpos=1&IdsFromResult=679582].

## Abbreviations

SMART: *Salmonella* Multiplex Assay for Rapid Typing
PCR: Polymerase Chain Reaction
WGS: Whole Genome Sequencing
LMICs: Low- and middle-income countries
NTS: Non-typhoidal *Salmonella*
ISO: International Organization for Standardization
CDC: Centers for Disease Control and Prevention
NGS: New Generation Sequencing
MDR: Multidrug Resistant
LaBESTA: Laboratoire de Biologie Moléculaire, d’épidémiologie et de surveillance des bactéries et virus transmissible par les aliments
U.S.: United States
USA: United States of America
